# Serum Albumin Levels as a Potential Marker for the Predictive and Prognostic Factor in Sudden Sensorineural Hearing Loss: A Prospective Cohort Study

**DOI:** 10.3389/fneur.2021.747561

**Published:** 2021-10-18

**Authors:** Zhong Zheng, Chengqi Liu, Ying Shen, Liang Xia, Lili Xiao, Yuanyuan Sun, Hui Wang, Zhengnong Chen, Yaqin Wu, Haibo Shi, Yanmei Feng, Shankai Yin

**Affiliations:** ^1^Department of Otolaryngology-Head and Neck Surgery, Shanghai Jiao Tong University Affiliated Sixth People's Hospital, Shanghai, China; ^2^Shanghai Key Laboratory of Sleep Disordered Breathing, Shanghai, China

**Keywords:** sudden sensorineural hearing loss, albumin, albumin-to-globulin ratio, predictive, prognostic

## Abstract

**Objectives:** As a common otology emergency, sudden sensorineural hearing loss (SSNHL) has a great impact on quality of life. The diagnosis and treatment of SSNHL remain challenging. This study aims to identify and investigate the association of liver functions with SSNHL.

**Methods:** A total of 135 SSNHL patients and 135 sex- and age-matched controls were prospectively enrolled. The baseline and clinical characteristics, along with liver function levels of participators, were collected. Linear correlation, logistic regression, and receiving operator characteristic curve analysis tests were applied to examine the association between liver function levels and SSNHL.

**Results:** There were no differences in baseline characteristics between SSNHL and control groups. The albumin (ALB) level of the SSNHL group was significantly lower than that in the control group (*p* < 0.001). The logistic regression showed that the low ALB level may be a predictive factor of SSNHL with an adjusted OR of 0.809 (95% CI, 0.742–0.882, *p* < 0.001). By comparing the indicators of different prognosis groups, we found that the effective group had a significantly lower hearing impair onset and higher ALB (*p* = 0.001) and AGR (*p* = 0.003) levels than the ineffective group. Logistic regression revealed that the hearing level onset (OR, 0.976; 95% CI, 0.956–0.997; *p* = 0.026) and ALB level (OR, 1.181; 95% CI, 1.071–1.301; *p* = 0.001) showed strong associations with treatment outcome.

**Conclusions:** Lower ALB levels, within the normal ranges, were associated with the occurrence and unfavorable outcome of SSNHL. However, further research on the underlying mechanisms needs to be conducted.

## Introduction

Sudden sensorineural hearing loss (SSNHL) has been defined as sudden hearing impairment at least 30 dB over at least three consecutive frequencies on audiogram within 3 days and with an uncertain cause (1). The yearly incidence of SSNHL is reported as 5–20 per 100,000 population, and it is estimated there are approximately 4,000 new cases per year in the USA (2, 3). Although the incidence rate of SSNHL in China has been increasing in recent years, the true incidence rate of SSNHL has not been well determined. While the etiology and pathophysiological mechanisms of SSNHL are not clear, microcirculation disorder, viral infection, and autoimmune disease have been implicated in its onset (1, 4). Previous studies have reported that the prognosis of SSNHL is related to factors such as initial time of treatment, audiogram shapes, and presence of vertigo (4). The guidelines of SSNHL in China and Germany suggest that different audiograms correlate with different causes of SSNHL. For the all frequencies of the SSNHL subtype (hearing impairment that occurred at all frequencies), hemodynamic disorder is a potential cause (4, 5). Since the inner ear is mainly supplied by a single terminal artery, microcirculation disturbance of the inner ear will have a serious impact on hearing given that the cochlear hair cells have high oxygen consumption and poor tolerance to hypoxia (6). Moreover, it is generally believed that the prognosis of all frequencies of the SSNHL subtype is poor (4), and patients are seriously affected with a low quality of life due to the irreversible hearing impairment.

The liver function test is an important index to evaluate the pathological changes of liver function, which is widely used in clinical diagnosis. It can reflect the metabolism, immunity, and damage repair ability of the body. The main indicators include albumin (ALB), globulin (GLB), alanine aminotransferase (ALT), aspartate aminotransferase (AST), and total bilirubin (TBIL). The physiological functions of ALB include maintaining osmotic pressure and transporting a variety of substances (7). The ALB level is often reduced in acute and chronic diseases, which not only provides strong independent information on the prognosis of diseases (8–10) but also has been a biomarker of inflammation (11, 12). Recently, increasing studies have confirmed that ALB plays an important role in the occurrence and development of cardiovascular and cerebrovascular diseases (13–15). Several studies indicated that SSNHL has similar pathogenesis with cardiovascular and cerebrovascular diseases (16–18). The determination of ALB values could be of prognostic value in acute inflammation and hemodynamic disorders related to SSNHL.

Given that liver function tests which include ALB are useful in assessing metabolism level, immune level, and damage repair level, observing the clinical data of SSNHL patients, we found that the liver function indexes, although within normal levels, had a certain trend in alteration. This may be related to the abnormal metabolism of the liver in the body and its influence on blood circulation. Therefore, the purpose of this prospective cohort study was to clarify the relationship between liver functions with SSNHL and to provide a theoretical basis for the prevention and treatment of SSNHL.

## Materials and Methods

### Study Population

A total of 135 consecutive patients with SSNHL diagnosed at our Hospital between July 2018 and December 2020 were prospectively enrolled. All participants provided written informed consent for their inclusion in the database and the use of their data for research purposes. The study protocol was approved by the Institutional Ethics Committee of the Shanghai Jiao Tong University Affiliated Sixth People's Hospital [2018-KY-036(K)]. This prospective cohort study was conducted in conformity with the Helsinki Declaration. Patients included in the study visited the hospital for the first time within 7 days after the onset of SSNHL. Based on the potential relationships between vascular factors and all frequencies SSNHL, we included patients with hearing loss at all frequencies and mean pure tone audiometry across 0.25 to 8 kHz ≥ 30 dBHL according to the American guideline of SSNHL (1). Patients were excluded from the study if they had any other otologic disease that can interfere with SSNHL diagnoses such as chronic otitis media, acoustic trauma or otologic surgery history, conductive hearing loss, Meniere's disease, and migraine. Recently experienced vomiting and diarrhea and a previous history of malignant disease, psychiatric conditions, or other major comorbidities (heart failure, stroke, hepatitis, and severe hepatic dysfunction) were also excluded. One hundred thirty-five sex- and age-matched controls with normal hearing and without any disease at regular health check-ups were included as a control group.

### Data Collection

Detailed medical history, which includes details of baseline characteristics [included age, sex, height, weight, body mass index (BMI), and blood pressure on admission (systolic blood pressure and diastolic blood pressure)] and clinical characteristics [included affected side, accompanying symptoms (like tinnitus, vertigo, ear fullness), time to treatment, and hearing level on admission], was obtained from all patients. All hearing assessments were performed in standard shielding rooms, and pure tone average (PTA) was performed for both air and bone conduction at 0.125, 0.25, 0.5, 1, 2, 4, and 8 kHz before and after 2 weeks of systemic treatment. The hearing level of each patient was calculated by averaging the PTA of impaired frequencies after onset while the extent of hearing recovery is calculated using PTA after onset minus PTA after treatment. Temporal bone computed tomography or inner ear magnetic resonance imaging was performed on all patients to rule out ear structural abnormality and tumors. Upon admission, liver function tests of ALB, GLB, ALT, AST, and TBIL levels were performed using blood samples obtained from the antecubital veins of all patients between 6 and 7 a.m. after an overnight fast. The liver function tests were assayed using an automatic biochemical analyzer (Hitachi 7600-120, Tokyo, Japan). The value of the albumin-to-globulin ratio (AGR) was obtained by the value of ALB divided by AGB.

### Treatment and Evaluation

The patients underwent comprehensive treatment, including treatment with steroids and batroxobin, according to the Chinese guideline of SSHNL (4). All patients were treated with a 7-day course of systemic glucocorticoid (prednisone 1 mg/kg/day for 3–5 days, followed by a reduced dosage for the remaining days according to the hearing improvement) and intravenous batroxobin (10 U batroxobin for the first time and then reduced to 5 U batroxobin, once every other day, one to three times in total according to the level of fibrinogen). Patients were classified into two groups according to their recovery in hearing observed in 2 weeks of follow-up: the effective group (PTA of impaired frequencies which improved more than or equal to 15 dB, or back to normal/unaffected ear) and the ineffective group (PTA of impaired frequencies which improved < 15 dB) (4).

### Statistical Analysis

All statistical analyses were performed using SPSS for Windows version 24.0 (IBM Corp., Armonk, NY, USA). First, the relationship assessment of liver functions with the occurrence of SSNHL was performed. Then, the relationship of liver functions with the occurrence of SSNHL was statistically investigated. Descriptive variables are expressed as mean ± standard deviation, median (interquartile range), or a percentage, as appropriate. Data were investigated using the Kolmogorov–Smirnov test to determine the distribution pattern. The data of age had a normal distribution (*p* > 0.05); thus, the comparisons of age were performed using the independent-sample *t*-test. The data of height, weight, BMI, body pressure, time to treatment, hearing level onset, and liver function levels did not have a normal distribution (*p* < 0.05); thus, Kruskal–Wallis test was used for the comparison of median values of the groups. The chi-squared test was used for categorical variables. A linear correlation was performed to assess the association between liver function levels, hearing loss, and hearing recovery. The backward conditional logistic regression (variables significant at *p* < 0.10 were considered for inclusion, and those significant at *p* < 0.05 were included) was used to estimate the odds ratios (OR) and 95% confidence intervals (CI) for the correlation between liver function levels and the occurrence and outcome of SSNHL. The collinearity of all continuous variables was examined before performing the logistic regression using the variance inflation factor. To detect the most significant parameter related to the outcome of SSNHL, and to determine a cutoff value, the receiver operating characteristic (ROC) curve analysis test was used. *p* < 0.01 was considered significant for all tests to account for small sample size. The figures were generated using GraphPad Prism 7.0 for Windows (GraphPad Software Inc., La Jolla, CA, USA).

## Results

### Baseline and Clinical Characteristics of Participants

A total of 135 SSNHL patients and 135 controls were enrolled. The baseline characteristics and liver function levels of participants are shown in Table 1. About half of the patients (*n* = 62, 45.93%) were male, and the mean age of the patients was 49.07 ± 16.44 years. There were no differences in age, sex distribution, height, weight, BMI, and blood pressure on admission between the two groups. Compared with the control group, the SSNHL had a significantly lower ALB level (*p* < 0.001). The levels of GLB, ALT, AST, and TBIL were not significantly different between the two groups (all *p* > 0.01).

**Table 1:** Baseline characteristics of participants in the SSNHL and control groups.

	**SSNHL (*n* = 135)**	**Control (*n* = 135)**	***p*-value**
**Baseline characteristics**			
Age (years)^*a*^	49.07 ± 16.44	47.10 ± 12.23	0.267
Sex (male, %)^*b*^	62 (45.93%)	65 (48.15%)	0.715
Height (cm)^*c*^	1.67 (1.61–1.74)	1.67 (1.61–1.74)	0.699
Weight (kg)^*c*^	66.00 (60.00–74.00)	64.90 (57.70–76.20)	0.836
BMI (kg/m^2^)^*c*^	23.89 (22.15–25.39)	23.80 (21.40–26.30)	0.721
Systolic blood pressure (mmHg)^*c*^	124.00 (112.00–132.00)	122.00 (117.00–129.00)	0.464
Diastolic blood pressure (mmHg)^*c*^	76.00 (71.00–85.00)	77.00 (71.00–80.00)	0.618
**Laboratory variables**			
ALB (g/L)^*c*^	45.00 (42.00–47.00)	47.00 (45.00–48.00)	<0.001^*^
GLB (g/L)^*c*^	26.00 (24.00–29.00)	27.00 (25.00–30.00)	0.685
AGR^*c*^	1.70 (1.43–1.88)	1.70 (1.55–1.92)	0.090
ALT (U/L)^*c*^	22.00 (13.00–31.00)	20.00 (17.00–24.00)	0.124
AST (U/L)^*c*^	19.00 (15.00–23.00)	18.00 (15.00–21.00)	0.544
TBIL (mg/dL)^*c*^	0.70 (0.53–0.88)	0.69 (0.67–0.79)	0.152

a *The values are given as mean ± standard deviation*.

b *The values are given as the number of cases and the percentage in parentheses*.

c *The values are given as median with its interquartile range (25–75th) in parentheses. SSNHL, sudden sensorineural hearing loss; BMI, body mass index; ALB, albumin; GLB, globulin; AGR, albumin-to-globulin ratio; ALT, alanine aminotransferase; AST, aspartate aminotransferase; TBIL, total bilirubin*.

* *The correlation was significant at the 0.01 level (p < 0.01)*.

### Liver Function Levels and the Occurrence of SSNHL

**Figure 1:**
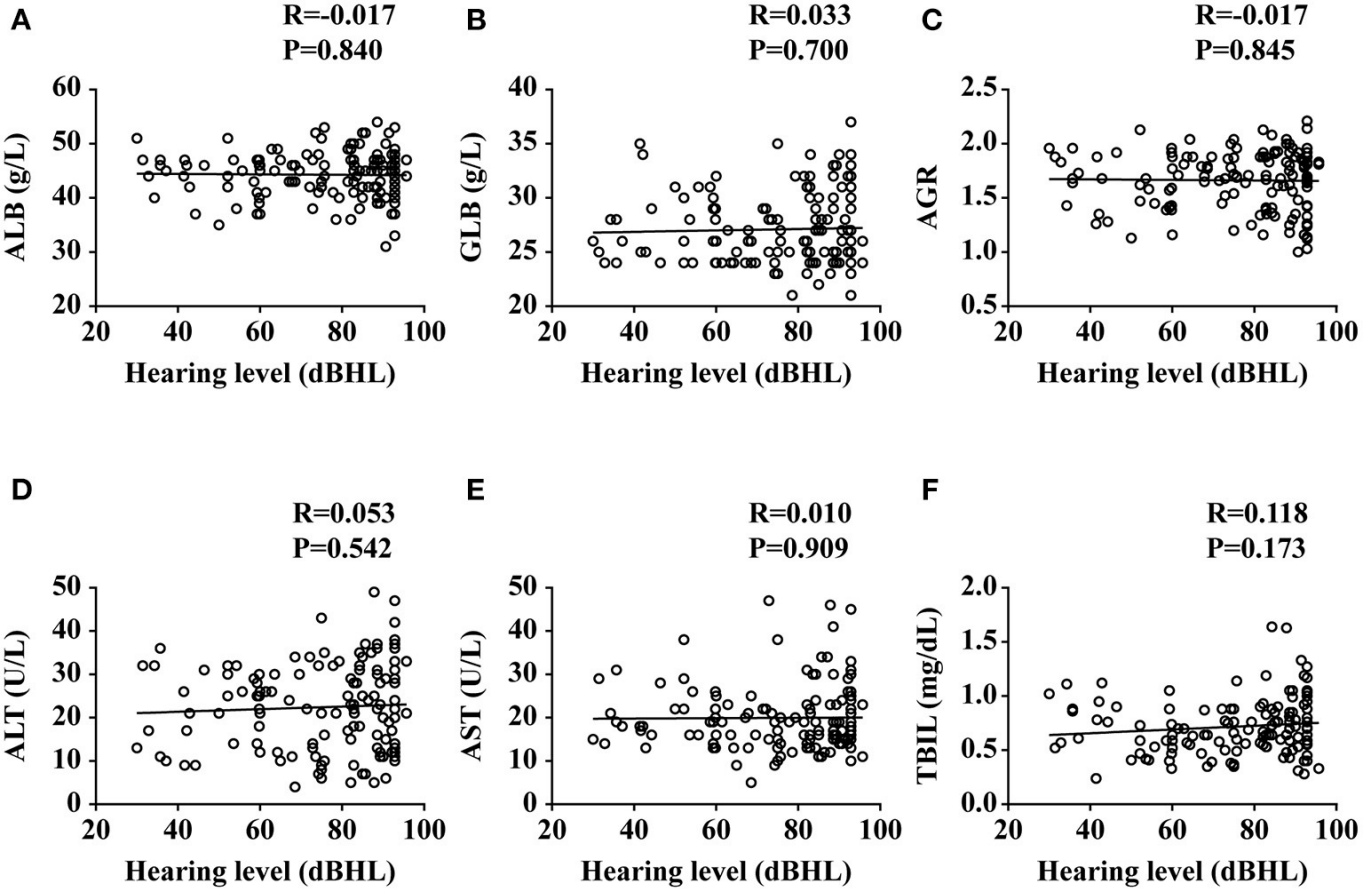
Plots of liver functions vs. the severity of hearing loss. ALB, albumin; GLB, globulin; AGR, albumin-to-globulin ratio; ALT, alanine aminotransferase; AST, aspartate aminotransferase; TBIL, total bilirubin. Data are presented as correlation coefficients (R) and *P* values. **(A–F)** represent the relationship of ALB, GLB, AGR, ALT, AST, and TBIL vs. the severity of hearing loss, respectively.

To investigate the relationship between liver function levels and the occurrence of SSNHL, we performed a linear correlation analysis and drew the scatterplots of liver function levels vs. severity of hearing loss, as shown in Figure 1, and found no association between liver function levels and SSNHL when hearing loss was treated as a continuous variable. This may suggest that the ALB level was not related to the severity of SSNHL. We then performed logistic regression models based on liver function levels and other parameters including age, sex, BMI, and body pressure, as shown in Table 2. The parameters of height, weight, and GLB level were eliminated by collinearity of all continuous variables using the variance inflation factor. The results show that the ALB level has a strong association with the occurrence of SSNHL with adjusted ORs of 0.809 (95% CI, 0.742–0.882, *p* < 0.001).

**Table 2:** Binary logistic regression analysis of the relationship between liver function levels and the occurrence of SSNHL.

**Parameter**	**Univariate analysis^a^**			**Multivariate analysis**		
	**OR**	**95% CI**	***p*-value**	**OR**	**95% CI**	***p-*value**
**Predictor: occurrence of disease**						
Age	1.005	0.986–1.024	0.599			
Sex (male)	1.039	0.616–1.752	0.885			
BMI	0.963	0.883–1.051	0.402			
Systolic blood pressure	1.019	0.991–1.048	0.185			
Diastolic blood pressure	0.995	0.955–1.037	0.811			
ALB level	0.805	0.727–0.892	<0.001*	0.809	0.742–0.882	<0.001*
AGR level	1.076	0.351–3.304	0.898			
ALT level	1.017	0.976–1.061	0.422			
AST level	1.022	0.967–1.080	0.434			
TBIL level	0.581	0.146–2.317	0.442			

a The parameters of height, weight, and GLB level were eliminated by collinearity of all continuous variables using the variance inflation factor. OR, odds ratio; CI, confidence interval; BMI, body mass index; ALB, albumin; GLB, globulin; AGR, albumin-to-globulin ratio; ALT, alanine aminotransferase; AST, aspartate aminotransferase; TBIL, total bilirubin. *The correlation was significant at the 0.01 level (p < 0.01).

### Liver Function Levels and the Functional Outcome of SSNHL

**Figure 2:**
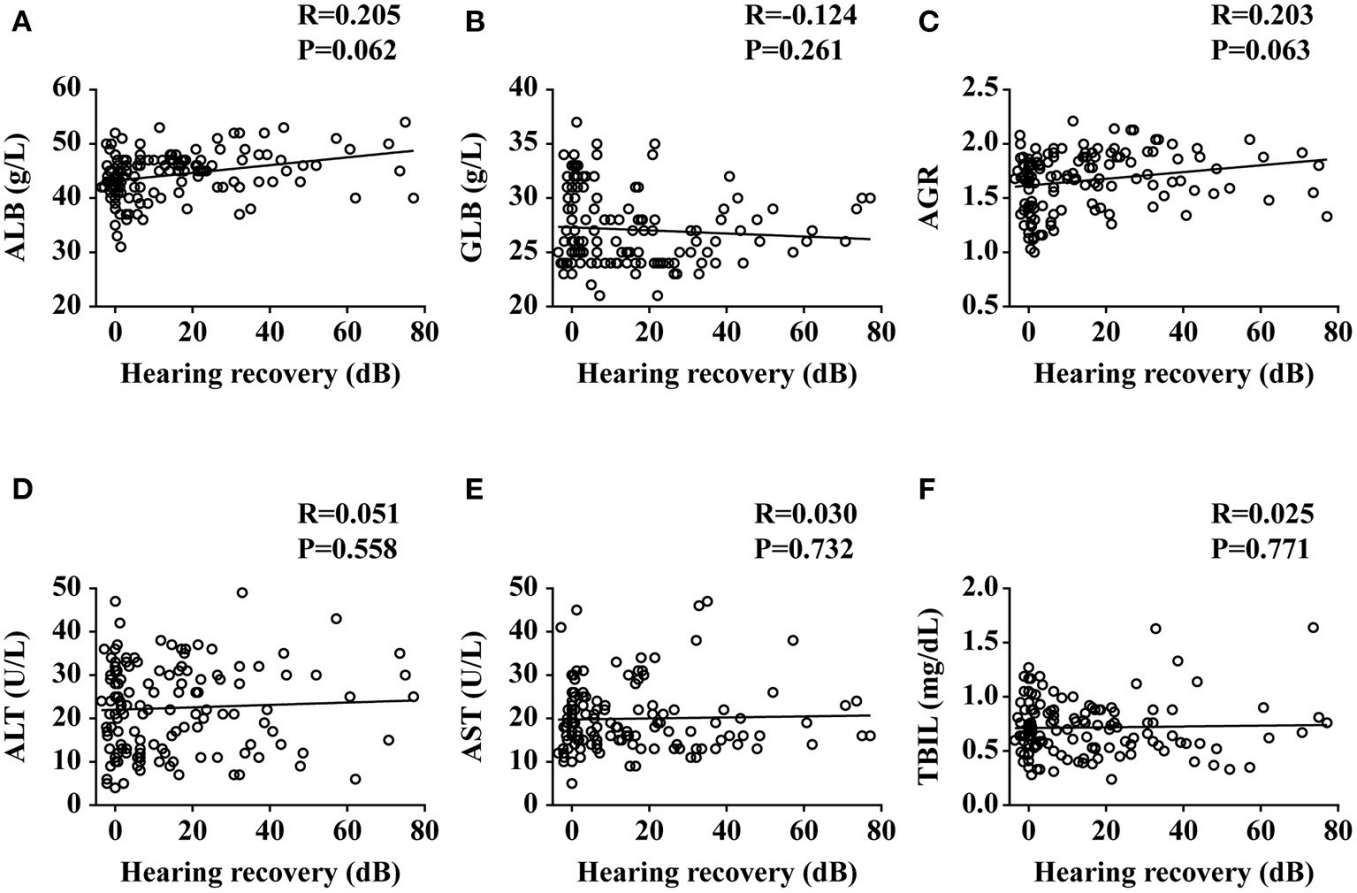
Plots of liver functions vs. the recovery of hearing level. ALB, albumin; GLB, globulin; AGR, albumin-to-globulin ratio; ALT, alanine aminotransferase; AST, aspartate aminotransferase; TBIL, total bilirubin. Data are presented as correlation coefficients (R) and *P* values. **(A–F)** represent the relationship of ALB, GLB, AGR, ALT, AST, and TBIL vs. the recovery of hearing level, respectively.

**Figure 3:**
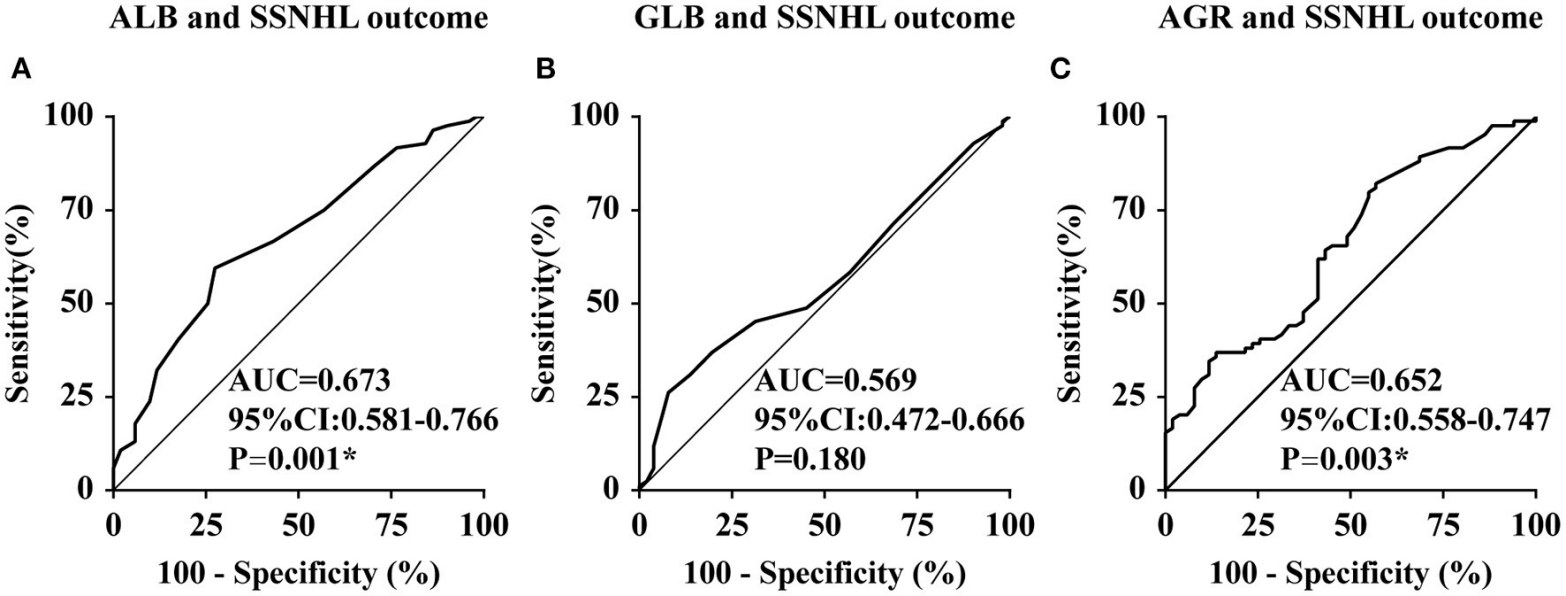
Receiver operating characteristic curve analysis of ALB, GLB, and AGR levels for the prediction of the outcome of SSNHL. ALB, albumin; GLB, globulin; AGR, albumin-to-globulin ratio; CI, confidence interval; SSNHL, sudden sensorineural hearing loss; AUC, area under the curve; CI, confidence interval. *The correlation was significant at the 0.01 level (*p* < 0.01). **(A–C)** represent the receiver operating characteristic curve analysis of ALB, GLB, and AGR levels for the prediction of the outcome of SSNHL, respectively.

The SSNHL patients were divided into the effective group (*n* = 51) and the ineffective group (*n* = 84) according to different levels of hearing recovery, as shown in Table 3. The baseline characteristics of the two groups had no significant difference (all *p* > 0.01). The clinical characteristics of the two groups had no significant difference (all *p* > 0.01), with the exception of hearing level being significantly higher in the ineffective group than that of the effective group (*p* = 0.007). Comparing the liver function levels of these two groups, we found that the effective group had significantly higher ALB (*p* = 0.001) and AGR (*p* = 0.003) levels than the ineffective group. We then performed a linear correlation analysis and drew the scatterplots of liver function levels vs. the recovery of hearing level, as shown in Figure 2, and found no association between liver function levels and SSNHL when hearing recovery was treated as a continuous variable. Although the levels of ALB and AGR were very close, it did not reach a level of significant difference (*p* = 0.062 and *p* = 0.063, respectively). Figure 3 shows the graph of the ROC curve analysis for the outcome of SSNHL; the areas under the curve were 0.673 (95% CI, 0.581–0.766), 0.569 (95% CI, 0.472–0.666), and 0.652 (95% CI, 0.558–0.747) for ALB, GLB, and AGR, respectively. The ROC curve analysis revealed that ALB and AGR levels may be imperfect prognostic predictors of SSNHL. Logistic regression models based on liver function levels and other parameters including age, sex, BMI, body pressure, affected side, tinnitus, vertigo, ear fullness, time to treatment, and hearing level onset are presented in Table 4. The parameters of height, weight, and GLB level were eliminated by collinearity of all continuous variables using the variance inflation factor. With unadjusted ORs of 0.970 (95% CI, 0.947–0.993, *p* = 0.012) and 1.191 (95% CI, 1.033–1.373, *p* = 0.016), the hearing level onset and ALB level showed a strong association with the treatment outcome. After adjusting for all other significant outcome predictors, the hearing level onset and ALB level remained independent outcome predictors with adjusted ORs of 0.976 (95% CI, 0.956–0.997, *p* = 0.026) and 1.181 (95% CI, 1.071–1.301, *p* = 0.001), respectively. These results showed that worse hearing level onset and lower ALB level correlate with worse SSNHL functional outcome.

**Table 3:** Demographics and laboratory variables in the SSNHL with different outcomes.

	**Effective (*n* = 51)**	**Ineffective (*n* = 84)**	***p*- value**
**Baseline characteristics**						
Age (years)^*a*^	49.20 ± 15.88	48.84 ± 17.47	0.903
Sex (male, %)^*b*^	25 (49.02%)	37 (44.05%)	0.574
Height (cm)	1.70 (1.62–1.77)	1.67 (1.61–1.72)	0.080
Weight (kg)	65.00 (60.00–75.00)	68.00 (60.00–72.00)	0.806
BMI (kg/m^2^)	23.89 (21.72–25.24)	23.90 (22.31–25.61)	0.533
Systolic blood pressure (mmHg)^*c*^	125.00 (113.00–133.00)	123.00 (111.25–131.75)	0.646
Diastolic blood pressure (mmHg)^*c*^	76.00 (71.00–86.00)	77.00 (69.50–81.75)	0.550
**Clinical characteristics**						
Affected side (left, %)^*b*^	25 (49.02%)	43 (51.19%)	0.807
Tinnitus (%)^*b*^	44 (86.27%)	70 (83.33%)	0.648
Vertigo (%)^*b*^	17 (33.33%)	21 (25.00%)	0.297
Ear fullness (%)^*b*^	13 (25.49%)	24 (28.57%)	0.697
Time to treatment (days)^*c*^	2.00 (2.00–3.00)	2.00 (2.00–4.00)	0.707
Hearing level (dBHL)^*c*^	75.00 (59.84–85.71)	84.29 (68.57–92.14)	0.007^*^
**Laboratory variables**						
ALB (g/L)^*c*^	46.00 (43.00–48.00)	43.50 (41.00–46.75)	0.001^*^
GLB (g/L)^*c*^	26.00 (24.00–28.00)	26.00 (24.00–31.00)	0.177
AGR^*c*^	1.80 (1.59–1.92)	1.68 (1.39–1.83)	0.003^*^
ALT (U/L)^*c*^	25.00 (14.00–31.00)	22.00 (13.00–30.75)	0.403
AST (U/L)^*c*^	18.00 (14.00–24.00)	19.00 (15.00–22.75)	0.815
TBIL (mg/dL)^*c*^	0.66 (0.53–0.88)	0.71 (0.54–0.88)	0.534

a *The values are given as mean ± standard deviation*.

b *The values are given as the number of cases and the percentage in parentheses*.

c *The values are given as median with its interquartile range (25–75th) in parentheses. BMI, body mass index; ALB, albumin; GLB, globulin; AGR, albumin-to-globulin ratio; ALT, alanine aminotransferase; AST, aspartate aminotransferase; TBIL, total bilirubin*.

* *The correlation was significant at the 0.01 level (p < 0.01)*.

**Table 4:** Binary logistic regression analysis of the relationship between liver function levels and the outcome of SSNHL.

**Parameter**	**Univariate analysis^a^**			**Multivariate analysis**		
	**OR**	**95% CI**	***p*-value**	**OR**	**95% CI**	***p-*value**
**Predictor: outcome of disease**						
Age	1.005	0.979–1.031	0.729			
Sex (male)	0.855	0.366–2.001	0.719			
BMI	0.980	0.846–1.135	0.786			
Systolic blood pressure	0.972	0.930–1.015	0.202			
Diastolic blood pressure	1.058	0.989–1.131	0.102			
Affected side (left)	1.085	0.452–2.604	0.854			
Tinnitus	0.371	0.111–1.235	0.106			
Vertigo	0.910	0.372–2.228	0.836			
Ear fullness	1.601	0.645–3.975	0.311			
Time to treatment	0.943	0.732–1.214	0.647			
Hearing level	0.970	0.947–0.993	0.012	0.976	0.956–0.997	0.026
ALB level	1.191	1.033–1.373	0.016	1.181	1.071–1.301	0.001^*^
AGR level	3.200	0.373–27.492	0.289			
ALT level	1.034	0.980–1.090	0.222			
AST level	1.019	0.952–1.092	0.586			
TBIL level	0.852	0.156–4.653	0.852			

a *The parameters of height, weight, and GLB level were eliminated by collinearity of all continuous variables using the variance inflation factor. OR, odds ratio; CI, confidence interval; BMI, body mass index; ALB, albumin; GLB, globulin; AGR, albumin-to-globulin ratio; ALT, alanine aminotransferase; AST, aspartate aminotransferase; TBIL, total bilirubin*.

* *The correlation was significant at the 0.01 level (p < 0.01)*.

## Discussion

The change in serum ALB level may be related to the prediction and prognosis of SSNHL. Given that serum ALB level may be related to hemodynamic disorders, we included SSNHL patients with hearing impairment at all frequencies in this study. The ALB level of SSNHL patients was significantly lower compared to the healthy control; meanwhile, the ALB and AGR levels of effective SSNHL were significantly greater compared with non-recovery SSNHL patients. Our results suggested that ALB might be the predictor parameter of SSNHL.

SSNHL is considered a significant medical emergency in otolaryngology practice. With the acceleration of social rhythm and the increasing pressure of life, the incidence of SSNHL has increased year by year and shows a trend of presenting in younger patients. The exact etiopathogenesis of SSNHL is still not fully determined. There are many hypotheses as to the origin of this disease: inflammation, viral infection, blood disorders, vascular causes, and immune disorders (19–21). With the development of research, some risk factors such as cardiovascular disease, hemorheology, and changes in high blood viscosity are also found to be associated with SSNHL (22, 23). Several multicenter studies reported that the type of hearing audiogram generated is one of the important factors affecting the prognosis of patients with SSNHL (4, 24), indicating different pathogenesis and different hearing outcomes. The hearing outcome of low-frequency SSNHL was found to be the best while all-frequency SSNHL is pessimistic (4, 24). Since the cochlea is mainly supplied by the labyrinthine artery (a single, terminal artery), the inner ear is susceptible to circulatory changes (25). The cochlea hair cells are extremely sensitive to ischemia and hypoxia, and hearing loss occurs during hemodynamic disturbance. Although there is no direct evidence that all-frequency SSNHL is caused by vascular disorder, Sun et al. (26) found that the MPV, a marker of atherosclerosis, in all-frequency SSNHL patients was significantly higher than that of the control group. Zheng et al. (27) performed vasodilation combined with glucocorticoid therapy for SSNHL based on possible thrombosis and vascular endothelial dysfunction and found that all-frequency SSNHL patients had a better prognosis with combined therapy. These factors combined suggest that all-frequency SSNHL is potentially related with inner ear circulation disorder.

ALB, commonly utilized to assess nutritional status, is an easily measured biomarker that correlates with clinical status. It is a cheap, easy-to-obtain, widely available clinical marker that can aid in the risk stratification of patients with various diseases (28). As a strong marker in diseases related to infection and inflammation, the ALB level usually decreases during the acute period (10, 29). Miraeidi et al. (12) reported that inflammation can lower ALB levels regardless of a patient's nutritional status. This conforms to our finding that the ALB and AGR levels decreased in the SSNHL group, and the lower ALB level may be a potential marker for the occurrence of SSNHL. A possible explanation for this phenomenon is that inflammatory mediators can promote ALB escape from capillaries and reprioritize liver protein synthesis in support of acute-phase reactants (30).

Recent studies have demonstrated that ALB takes an active role in the pathophysiology of thrombosis, coagulation, and atherosclerosis (13–15). A low ALB level, even within the normal range, is associated with myocardial infarction, coronary heart disease, and stroke morbidity and mortality (13, 15, 31, 32). This study found that ALB levels of the effective group were significantly higher than those in the ineffective group, and ALB may be a strong marker for a favorable outcome of SSNHL.

Although the exact mechanism is not clear, we speculate that several biologic mechanisms might explain these associations. First, the ALB can bind to several ligands (such as nitric oxide) and interact with free fatty acids that inhibit their promoting effects on vascular tension and inhibit platelet aggregation and thrombosis (33, 34). The disturbance of the inner ear microcirculation of SSNHL can be greatly alleviated. Second, the ALB acts as an indirect and sacrificial antioxidant (35, 36) that can inhibit peroxidase and free radical generation (36). It should be noted that oxidative stress may also be involved in the pathogenesis of SSNHL (37). Third, the ALB is an inhibitor of human endothelial cell apoptosis (38) and can also help maintain endothelial permeability *via* interactions with the interstitial matrix (39). Endothelial dysfunction has been described in patients with SSNHL, which supports the pathogenesis of vascular involvement in SSNHL. Endothelial dysfunction may also lead to impaired labyrinthine perfusion and reduced hearing capability in SSNHL patients (40). Finally, the ALB can elevate serum osmotic pressure, which attracts interstitial fluid and improves organ perfusion (29). Hearing recovery is thus facilitated by improved perfusion of the inner ear.

GLB is composed of a number of inflammation-related proteins (such as complement, interleukin-6, and immunoglobulin). Thus, the increase in GLB reflects an inflammatory state (41). Since both reduced ALB and increased GLB play an important role in inflammation, the AGR can accurately indicate the inflammatory state of the body and many diseases can influence the AGR level. Several studies have indicated that the levels of inflammatory factors (e.g., C-reactive protein, erythrocyte sedimentation rate, and neutrophil/lymphocyte ratio) were elevated in SSNHL (26, 42–44). In our research, we found that the AGR level is significantly higher in the effective group than in the ineffective group. This may indicate that a high AGR level may lead to a better hearing outcome.

Recent studies indicated that TBIL is a potent antioxidant with anti-inflammatory properties *in vivo* and *in vitro* (45). Bing et al. (46) reported that higher TBIL levels, within the normal or mildly elevated ranges, were independently associated with better outcomes in bilateral SSNHL patients in hearing outcome measures such as final hearing threshold and absolute and relative hearing gains. Unlike this study, our research found no relationship between TBIL and SSNHL; this may be caused by the different subtypes of selected SSNHL patients.

To our knowledge, this is the first study to clarify the potential beneficial effect of ALB on occurrence and hearing outcomes in SSNHL patients. Given the inflammatory and microcirculation basis of the pathogenesis of SSNHL, it may be logical to assess ALB before initiating treatment in order to predict the prognosis of hearing loss. In addition, it proposes a new therapeutic target for patients with SSNHL. However, this study has some limitations. Firstly, this was a single-center study with a small sample size, and the results were susceptible to selection bias, even though we used strict standard inclusion and exclusion criteria. Secondly, the ALB level was measured only on admission, which made it impossible to examine its change over time. Thirdly, the follow-up time is short. Yeo et al. (47) did a retrospective study that included 156 patients who were treated by a 10-day course of admission therapy and followed for at least 3 months. They found that 54.5% patients recovered within that 10-day period, and of the other 45.5% patients, 78.2% recovered within 1 month while 21.8% patients had delayed hearing recovery later than 1 month after discharge. This indicated that a longer follow-up time is needed. Lastly, the strength of the association between ALB with hearing impairment and hearing outcome was not satisfactory; this may indicate that there are other factors at work. Further basic research is needed to elucidate the mechanism between ALB levels and SSNHL incidence.

## Conclusion

In the present study, we investigated the occurrence and hearing outcome in SSNHL patients in relation to serum liver function levels. The ALB level is positively associated with hearing outcomes according to PTA of impaired frequencies. Better hearing level onset and higher ALB level may also lead to favorable SSNHL functional outcomes. However, these factors alone could not completely determine the onset of and recovery from SSNHL, and further basic and clinical studies are needed for confirmation.

## Data Availability Statement

The raw data supporting the conclusions of this article will be made available by the authors, without undue reservation.

## Ethics Statement

The studies involving human participants were reviewed and approved by Ethics Committee of the Sixth People's Hospital affiliated to the Shanghai Jiao Tong University. The patients/participants provided their written informed consent to participate in this study.

## Author Contributions

ZZ, CL, HS, and YF: designed and supervised the research. CL: analyzed the data. YSh, LXia, LXiao, and YSu: gave suggestions on the data acquisition and analysis. ZZ: wrote the manuscript. HW, ZC, YW, HS, and SY: participated in manuscript editing. YF: support for this assistance was funded. All authors reviewed the manuscript.

## Funding

This study was supported by the National Natural Science Foundation of China (81771015), the First Grant (2020YFC2005201) of the Chinese National Key Research and Development Program (2020YFC2005200), and the Shanghai Municipal Commission of Science and Technology (Grant No. 18DZ2260200).

## Conflict of Interest

The authors declare that the research was conducted in the absence of any commercial or financial relationships that could be construed as a potential conflict of interest.

## Publisher's Note

All claims expressed in this article are solely those of the authors and do not necessarily represent those of their affiliated organizations, or those of the publisher, the editors and the reviewers. Any product that may be evaluated in this article, or claim that may be made by its manufacturer, is not guaranteed or endorsed by the publisher.
